# A retrospective study of vector‐borne disease prevalence in dogs with proteinuria: Southeastern United States

**DOI:** 10.1111/jvim.15610

**Published:** 2020-01-08

**Authors:** Emily K. Purswell, Erin W. Lashnits, Edward B. Breitschwerdt, Shelly L. Vaden

**Affiliations:** ^1^ The Department of Clinical Sciences and North Carolina State University College of Veterinary Medicine, North Carolina State University Raleigh North Carolina

**Keywords:** anaplasmosis, ehrlichiosis, flea, immune‐mediated, rickettsioses, tick

## Abstract

**Background:**

Proteinuria is a risk factor for progressive kidney injury in dogs. Enhanced understanding of potential associations between canine vector‐borne diseases (CVBD) and proteinuria is needed.

**Objectives:**

To determine the proportion of evaluated proteinuric dogs exposed to ≥1 CVBD, including *Babesia* spp., *Ehrlichia* spp., spotted‐fever group *Rickettsia*, *Bartonella* spp., *Anaplasma* spp., hemotropic *Mycoplasma* spp., *Borrelia burgdorferi*, and *Dirofilaria immitis*, and to determine if demographic or clinicopathologic differences exist between proteinuric dogs exposed to CVBD versus proteinuric dogs with no evidence of CVBD exposure.

**Animals:**

Two‐hundred nine proteinuric dogs, concurrently tested for CVBD, which were examined at a single academic veterinary hospital between January 2008 and December 2015.

**Methods:**

Retrospective cross‐sectional study. Demographic, clinicopathologic, and CVBD test results were extracted from medical records. A multivariable logistic regression model was used to assess associations between CVBD and selected variables.

**Results:**

Based on serology and polymerase chain reaction testing, 34% of proteinuric dogs (72/209) were exposed to ≥1 CVBD. Exposure to *Rickettsia* spp. (19%), *Ehrlichia* spp. (12%), and *B*. *burgdorferi* (9%) were most common. The CVBD exposure was lower in dogs tested in autumn or spring, higher in intact dogs, and higher in dogs with lower serum albumin and higher serum creatinine concentrations.

**Conclusions and Clinical Importance:**

Exposure to CVBD, particularly exposure to *Rickettsia* spp., *Ehrlichia* spp., and *B*. *burgdorferi* was found in proteinuric dogs from the southeast United States. Additional controlled prospective studies examining a potential causal relationship between CVBD and proteinuria are warranted.

**Abbreviations**
CI: confidence interval
CVBD: canine vector‐borne disease
GOF: goodness of fit
HCT: hematocrit
IFA: indirect immunofluorescent assay
IMGN: immune‐mediated glomerulonephritis
OR: odds ratio
P: proteinuria
PCR: polymerase chain reaction
SFG: spotted fever group
UPC: urine protein:creatinine

## 1 INTRODUCTION

Proteinuria is an important clinical finding and is not only a marker of nephropathy but also a prognostic factor for progression of chronic kidney disease.^1^, ^2^, ^3^, ^4^, ^5^, ^6^, ^7^, ^8^ Proteinuria is related to decreased survival in azotemic and non‐azotemic dogs, and decreasing proteinuria improves survival.^1^, ^2^, ^4^, ^9^ Because a variety of disease processes can cause or contribute to proteinuria, not all of which can be treated, it is important to identify opportunities for clinical interventions that may prevent progression of kidney injury in proteinuric dogs. Because of the possibility for chronic persistence of canine vector‐borne disease (CVBD) pathogens within the bloodstream or tissues, the potential role of CVBD as a cause of, or contributor to, proteinuria in dogs requires investigation.

Several CVBDs have been associated with proteinuria and immune‐mediated glomerulonephritis (IMGN) in dogs, including *Anaplasma* spp., *Bartonella* spp., *Borrelia burgdorferi*, *Babesia canis*, *Ehrlichia canis*, *Hepatozoon americanum*, and *Leishmania infantum*.^10^, ^11^, ^12^, ^13^, ^14^, ^15^, ^16^, ^17^ The most common cause of glomerular disease in dogs, IMGN, is caused by deposition of immune complexes in the glomerular capillary walls that disrupt the glomerular filtration barrier and lead to proteinuria.^18^ The exact nature of the antigens inciting immune complex formation rarely is determined. Clinicians attempting to prevent disease progression may elect to treat proteinuric dogs with suspected or confirmed glomerular disease based on indirect evidence of comorbidities that may be leading to immune complex formation, such as positive serology for CVBD.

When searching for an underlying infectious disease as a component of the evaluation for proteinuric dogs, clinicians are advised to use their clinical judgment and be cognizant of potential CVBD exposures that may be unique for each patient.^10^, ^18^ Although associations between IMGN and certain CVBDs have been reported, the prevalence of, and risk factors for, infection with these organisms in dogs with proteinuria have remained largely unstudied. Epidemiologic studies of CVBD exposure in proteinuric dogs can provide important information on the prevalence of diseases in certain demographic groups and locations, and the seasons when transmission or disease expression are most common. Additionally, although coinfection with multiple vector‐borne pathogens can cause more severe disease manifestations, this association has not been investigated specifically for proteinuric dogs.^19^, ^20^

The purpose of our retrospective study was to describe the CVBD exposures among proteinuric dogs. The first aim was to determine the proportion of evaluated proteinuric dogs exposed to each CVBD of interest, including *B canis*, *Babesia gibsoni*, *E canis*, spotted‐fever group *Rickettsia*, *Bartonella vinsonii* spp. *berkhoffii*, *Bartonella henselae*, *Bartonella koehlerae*, *Anaplasma* spp., hemotropic *Mycoplasma* spp., *B burgdorferi* (Lyme disease), and *Dirofilaria immitis* (heartworm disease). The second aim was to determine if demographic or clinicopathologic differences existed between proteinuric dogs exposed to CVBD (P‐CVBD+) and proteinuric dogs that lacked serological or polymerase chain reaction (PCR) evidence of CVBD exposure (P‐CVBD−). Our hypothesis was that there would be no significant differences in demographics or clinicopathologic variables between these groups.

## 2 MATERIALS AND METHODS

### 2.1 Setting and participants

**Figure 1:**
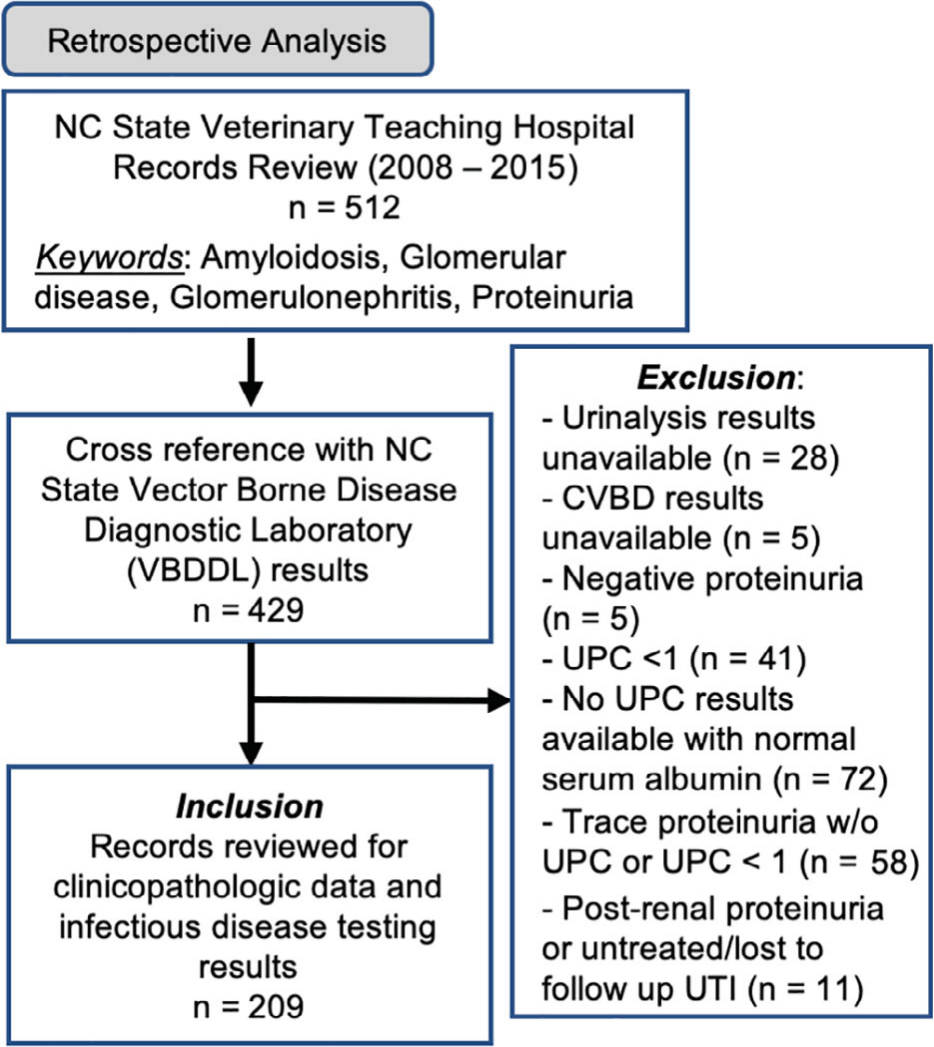
Inclusion and exclusion criteria

To identify cases for this retrospective cross‐sectional study, medical records for dogs presenting to NC State University Veterinary Hospital (NCSU‐VH) from January 2008 to December 2015 were searched using keywords: amyloidosis, glomerulonephritis, glomerular disease, and proteinuria. The results of this search then were cross‐referenced with the NCSU Vector Borne Disease Diagnostic Laboratory (VBDDL) database to identify dogs that had ≥1 concurrent (within 30 days) CVBD test results indirect immunofluorescent assay [IFA] or PCR. Complete medical records for each dog identified by keyword search with concurrent CVBD testing were reviewed to confirm inclusion. Dogs were excluded using a multistep process (Figure 1). All dogs were required to have a urinalysis performed within 1 month of CVBD testing; if no urinalysis result was available dogs were excluded. All dogs were required to have a urine protein:creatinine (UPC) ratio ≥1, or if no UPC result was available, they were required to have ≥1 proteinuria on urinalysis dipstick evaluation and concurrent hypoalbuminemia or otherwise were excluded. Dogs with urinary tract infection (bacteriuria and pyuria on urinalysis, positive urine culture or both) were excluded. One dog was included in the study despite having culture‐positive urinary tract infection at the time of testing because it had a renal biopsy performed and was confirmed to have IMGN. Dogs also were excluded if immunosuppressive drugs were being administered at the time of diagnostic testing. Prior antibiotic treatment was not an exclusion criterion.

For comparison with the proteinuric dog study group, the prevalence of each CVBD in the overall population of dogs tested by the VBDDL for CVBD from the same region and time period was calculated. Prevalence was calculated for 2 populations: the VBDDL NCSU referral population, composed of all dogs' samples submitted to the VBDDL for any CVBD IFA or PCR test from patients of the NCSU Veterinary Hospital between January 1, 2008, and December 31, 2015, (n = 2661), and the VBDDL southeast population, composed of all dogs' samples submitted to the VBDDL for any CVBD IFA or PCR test from veterinary clinics located in the southeast United States (North Carolina, South Carolina, Virginia, or Tennessee) between January 1, 2008, and December 31, 2015 (n = 7202).

### 2.2 Variables and data sources

When available, the following information was extracted from the medical records of the 209 proteinuric dogs included in the study: age, sex, breed, clinicopathologic data (CBC, serum biochemistry profile, urinalysis, and UPC ratio) and CVBD serology and PCR test results. Complete blood count, serum biochemistry profiles, urinalysis, and UPC ratios were performed by the clinical pathology laboratory at NCSU. For the purpose of statistical analyses, dogs that met the inclusion criteria were separated into 2 groups: proteinuric dogs with ≥1 serological or PCR‐positive CVBD test results indicating CVBD exposure (P‐CVBD+) and proteinuric dogs with no serological or PCR‐positive CVBD test results (ie, every CVBD test result was negative), indicating no CVBD exposure (P‐CVBD−).

The specific pathogens on the CVBD serology and PCR panel assays offered by the NCSU VBDDL during the study period included *B canis*, *B gibsoni*, *E canis*, spotted‐fever group *Rickettsia*, *B vinsonii* spp. *berkhoffii*, *B henselae*, *B koehlerae*, *Anaplasma* spp., hemotropic *Mycoplasma* spp., *B burgdorferi* (Lyme disease), and *D immitis* (heartworm disease). Over the 7‐year study period, not all samples were tested for all pathogens based on clinician discretion, changes in pathogen testing capabilities by the NCSU VBDDL, or both.

Serum samples included in the study were submitted by the attending clinician to the NCSU VBDDL for individual serological tests or for a comprehensive vector‐borne pathogen serology panel. Serum samples were tested using IFA assays for *B canis*, *B gibsoni*, *B henselae*, *B vinsonii* spp. *berkhoffii*, *E canis*, and *R rickettsia* antibodies and for *D immitis* antigen, and *Anaplasma* spp. (*A phagocytophilum* and *A platys*), *Ehrlichia* spp. (*E canis* and *E chaffeensis*), and *B burgdorferi* antibodies using a commercial ELISA‐based assay (SNAP 4Dx, IDEXX Laboratories, Inc, Westbrook, Maine). Between January 2008 and July 2011, only *B henselae* and *B vinsonii* spp. *berkhoffii* were used as antigens for IFA testing. After July 2011, the serology panel was amended to include *B koehlerae*. Before July 2012, comprehensive panels included a SNAP 4Dx (IDEXX Laboratories); starting in July 2012, this was changed to a SNAP 4Dx PLUS test. With the development and the use of the SNAP 4Dx PLUS assay, an *Ehrlichia ewingii* peptide was added, providing enhanced sensitivity for detection of antibodies to this *Ehrlichia* sp. With SNAP assays, ELISA antibody cross‐reactivity occurs with *A phagocytophilum* and *A platys* and with *E canis* and *E chaffeensis*.^21^, ^22^, ^23^ All NCSU VBDDL IFA antigens were grown in vitro or, in the case of *B canis*, in vivo by personnel in the VBDDL using bacterial or protozoan strains of canine or feline origin. Seroreactive samples were defined as having end point IFA titers ≥1:64. By IFA testing, serological cross‐reactivity occurs between *R rickettsii* and other spotted fever group (SFG) rickettsiae, including *R parkeri*, *R felis*, *R amblyommi*, and other seemingly nonpathogenic SFG rickettsiae often found in ticks. Therefore, IFA and ELISA seroreactivity, as reported in our study, indicate exposure to *Anaplasma*, *Ehrlichia*, or SFG *Rickettsia* spp. *Ehrlichia* IFA and ELISA reactivity were combined to represent exposure ≥1 *Ehrlichia* spp. Neither IFA nor ELISA assays distinguish between acute and chronic infections, unless seroconversion is documented.^21^, ^22^, ^23^

Polymerase chain reaction for *Bartonella* intergenic transcribed spacer region was performed targeting the region between the *Bartonella* 16S‐23S ribosomal RNA genes. Primers and PCR conditions have been described previously.^24^, ^25^ Similarly, primers and PCR conditions for *Babesia* spp., hemotropic *Mycoplasma* spp., *Rickettsia* spp., *Ehrlichia*, and *Anaplasma* were used as previously described.^26^, ^27^, ^28^ All PCR‐positive amplicons were sequenced, and consensus sequences were aligned (Vector NTI Suite 10.1, Invitrogen Corp, California) with known sequences in GenBank using the basic local alignment search tool (BLAST), available from (http://www.ncbi.nlm.nih.gov/BLAST/). Previously described negative and positive controls were used for each PCR assay.

### 2.3 Statistical analysis

Descriptive statistics were obtained for proteinuric dogs with and without CVBD exposure as defined by the previously described testing methods. The Shapiro‐Wilk test for normality was used, and continuous variables with normal distribution were described using mean and standard deviation, whereas nonnormally distributed continuous variables were described using median and interquartile range. Differences between P‐CVBD+ and P‐CVBD− groups were calculated using *t* tests for continuous normal variables (age, hematocrit [HCT], serum albumin concentration), Wilcoxon rank‐sum test with continuity correction for continuous nonnormal variables (platelet and lymphocyte counts, serum creatinine, globulin, and cholesterol concentrations; and UPC), and chi‐squared tests (or Fisher's exact tests for small sample sizes) for categorical variables (breed, sex, season of admission, coinfection). Differences in demographic factors and clinicopathologic variables among levels of coinfection were assessed using chi‐squared tests or Kruskal‐Wallis tests, respectively. Differences in the proportion of dogs exposed to each CVBD between the proteinuric dog study sample compared to the VBDDL NCSU referral population and the VBDDL southeast population were calculated using chi‐squared tests (or Fisher's exact tests for small sample size).^23^, ^27^, ^28^

To determine if CVBD exposure was associated with any particular clinicopathologic findings, associations between each selected clinicopathologic variable and P‐CVBD+ versus P‐CVBD− groups first were assessed separately using univariable logistic regression. The outcome variable included dogs that were either seroreactive or PCR positive (or both) for ≥1 CVBD. Factors with *P* < .25 in a univariable regression were considered for inclusion in a multivariable logistic regression model (breed, intact status, season of admission, platelet count, serum albumin concentration, serum creatinine concentration, serum cholesterol concentration, and UPC ratio).^29^ The multivariable model was constructed using a stepwise approach. Pearson's correlation coefficients were used to test for collinearity of explanatory variables and to determine whether to include variables that appeared to be correlated. Explanatory variables with plausible biological associations were tested for interactions. The most parsimonious multivariable model was selected, and goodness of fit (GOF) was assessed using the Hosmer‐Lemeshow GOF test. Correlations between numerical clinicopathologic variables were calculated using Pearson's correlation coefficients (*ρ*). A multivariable logistic regression model for a secondary outcome of interest, *B burgdorferi* exposure, also was created using the same methodology and explanatory variables, including demographic factors (age, breed, and sex) as well as season of presentation. Analyses were performed in R 3.3.1^30^ using the “MASS,”^31^ “Resource Selection,”^32^, ^33^, ^34^ and “corrplot”^35^ packages. Statistical significance was considered at a *P* value of ≤.05, except where otherwise indicated.

## 3 RESULTS

During the 2008‐2015 study period, 429 medical records met the initial search criteria, of which 209 met the final proteinuria inclusion criteria. During the 7‐year study period, the largest number of cases was found in 2011 (34, 16.3%) and the smallest number in 2012 (19, 9.1%). The breeds most frequently represented in the study population included Labrador Retrievers (25, 12.0%), mixed breed dogs (20, 9.6%), and Golden Retrievers (14, 6.7%), with dogs from each remaining 64 breeds making up <5% of the overall study population (Table 1). Dogs' age ranged from 9 months to 16 years, with a mean of 8.1 years; age was not reported for 1 dog. There were 104 males (80 neutered, 77%) and 105 females (103 spayed, 98%).

**Table 1:** Demographic and clinicopathologic characteristics of study population

	All		P‐CVBD−		P‐CVBD+		
	Mean/median	SD/range	Mean/median	SD/range	Mean/median	SD/range	*P*‐value
Age	8.11	3.25	8.12	3.46	8.10	2.84	.96
Alb	2.6	0.69	2.67	0.72	2.46	0.6	.04*
HCT	36.09	11.04	36.23	11.06	35.8	11.1	.80
Chol	269.5	78‐1405	277.5	108‐1405	245	78‐555	.06
Creat	0.9	0.2‐12.9	0.8	0.2‐8.5	1.2	0.2‐12.9	.11
Glob	2.8	1.5‐8.3	2.9	1.5‐8.3	2.75	1.6‐6.2	.29
LC	1.01	0‐14.57	0.99	0‐14.57	1.07	0‐3.4	.85
Plt	271	1‐895	296	1‐895	230.5	2‐696	.10
UPC	3.93	1‐34.89	3.9	1‐25.98	5.03	1.01‐34.89	.10

	Number (%) of each		Number (%) of each		Number (%) of each		
*Sex*							.02*
FI	2 (1.0)		0 (0)		2 (100)		
FS	103 (45.0)		74 (54.0)		29 (40.3)		
MI	24 (11.5)		11 (8.0)		13 (18.1)		
MN	80 (38.3)		52 (38.0)		28 (38.9)		
*Breed*							.51
Lab	25 (12.0)		18 (13.1)		7 (9.7)		
Golden	14 (6.7)		7 (5.1)		7 (9.7)		
Mix	20 (9.6)		12 (8.8)		8 (11.1)		
Other	150 (71.8)		100 (73.0)		50 (69.4)		
*Season*							.01*
Autumn	51 (24.4)		38 (27.7)		13 (18.1)		
Winter	51 (24.4)		28 (20.4)		23 (31.9)		
Spring	49 (23.4)		39 (28.5)		10 (13.9)		
Summer	58 (27.8)		32 (23.4)		26 (36.1)		

Mean and standard deviation shown for normally distributed data (age, Alb, HCT); median and range shown for skewed data (Chol, Creat, Glob, LC, Plt, UPC).

Abbreviations: CVBD, canine vector‐borne disease; HCT, hematocrit; LC, total lymphocyte count; P, proteinuria; P‐CVBD−, proteinuric dogs lacking serological or polymerase chain reaction (PCR) evidence of exposure to CVBD; P‐CVBD+, proteinuric dogs with serological or PCR evidence of exposure to CVBD; SD, standard deviation; UPC, urine protein:creatinine ratio.

**Figure 2:**
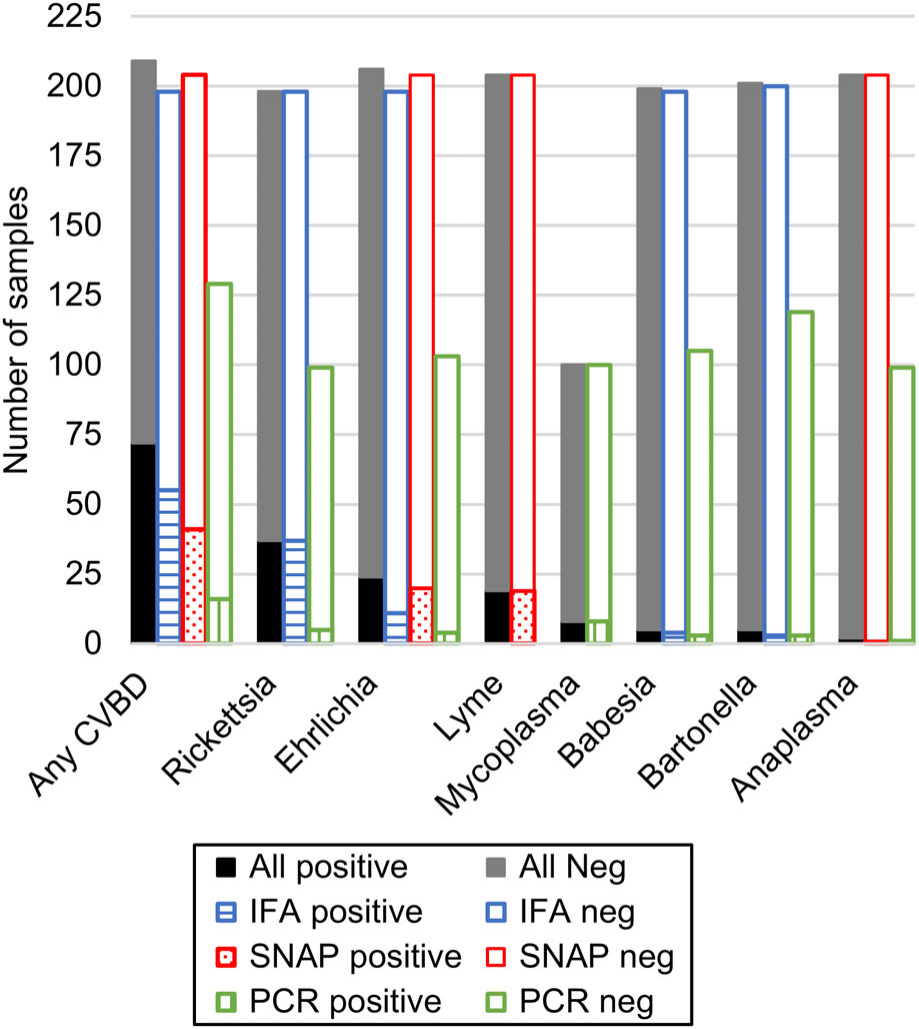
Results of canine vector‐borne disease testing. Each column represents the number of samples tested with each method for each pathogen. Gray scale column represents the combination of all testing methods for each pathogen, with positive tests in black and negative tests in gray. The colored bars represent each testing type, with the positive tests shaded and the negative tests white (Blue = IFA, red = SNAP, and green = PCR)

On the basis of serology or PCR testing, 34.4% of proteinuric dogs (72/209) were exposed to ≥1 CVBD (Figure 2, Table 2). Exposure to *Rickettsia* spp. (37/198, 18.7%), *Ehrlichia* spp. (24/206, 11.7%), *B burgdorferi* (19/204, 9.3%), and hemotropic *Mycoplasma* spp. (7/99, 7.1%) occurred most often. *Babesia* spp., *Bartonella* spp., *Anaplasma* spp., and *D*. *immitis* were found in ≤5 dogs either by serology or PCR testing modalities (Table 2). Of the 209 dogs, serology results were available for 208 and PCR results for 128. Of the 128 dogs tested by PCR, 1 dog was infected with 2 pathogens (*R rickettsii* and “*Candidatus Mycoplasma haematoparvum*”), and 21 dogs (16.4%) with 1 pathogen; the remaining 106 dogs (82.8%) had negative PCR test results. The SNAP 4DX or 4DX PLUS ELISA results were available for 204 of the 209 proteinuric dogs, of which 20 (9.8%) were *Ehrlichia* spp. positive, 19 (9.3%) *B*. *burgdorferi* positive, and 1 each *Anaplasma* spp., and *D*. *immitis* positive. Of the 172 dogs with negative SNAP ELISA results, 39 (22.7%) were P‐CVBD+, including 2 dogs seroreactive on IFA for *E canis* and 37 dogs positive for pathogens not included on the SNAP 4DX ELISA (Table 3).

**Table 2:** Results from CVBD testing of proteinuric dogs compared to the VBDDL‐NCSU referral population and the VBDDL southeast population during the study period

	Proteinuric dogs		VBDDL NCSU referral			VBDDL southeast		
	Positive (%)	Total tested	Positive (%)	Total tested	*P*‐value (versus RPG)	Positive (%)	Total tested	*P*‐value (versus RPG)
Any	72 (34.4)	209	689 (25.9)	2661	.009*	1734 (24.1)	7202	<.001*
*Rickettsia* total	37 (18.7)	198	301 (13.3)	2258	.05*	691 (13.3)	5198	.03*
IFA	37 (18.7)	198	298 (13.3)	2244	.04*	680 (13.5)	5050	
PCR	5 (5.0)	99	4 (0.6)	668	<.001*	15 (1.2)	1296	
*Ehrlichia* total	24 (11.7)	206	323 (13.6)	2379	.50	717 (12.6)	5672	.75
SNAP + IFA	24 (11.7)	206	307 (13.6)	2257		640 (12.6)	5092	
PCR	4 (3.9)	103	32 (3.9)	822		105 (5.7)	1828	
Lyme (SNAP)	19 (9.3)	204	81 (3.6)	2229	<.001*	269 (5.4)	4953	.03*
								
*Mycoplasma* (PCR)	7 (7.1)	99	20 (3.0)	667	.08	41 (3.3)	1233	.04*
*Babesia* total	5 (2.5)	199	43 (1.8)	2373	.67	133 (2.3)	5722	.84
IFA	4 (2.0)	198	25 (1.1)	2222		62 (1.2)	5164	
PCR	3 (2.9)	105	26 (2.8)	945		88 (4.6)	1899	
*Bartonella* total	5 (2.5)	201	51 (2.1)	2402	.93	159 (2.6)	6103	.92
IFA	3 (1.5)	200	45 (2.0)	2262		145 (2.6)	5593	
PCR + BAPGM	3 (2.5)	119	7 (0.8)	851		18 (1.0)	1814	
*Anaplasma* total	2 (1.0)	204	21 (0.9)	2268	.77	75 (1.5)	5069	.77
SNAP	1 (0.5)	204	16 (0.7)	2229		67 (1.4)	4886	
PCR	1 (1.0)	99	6 (0.9)	700		10 (0.7)	1346	
HW (SNAP)	1 (0.5)	204	19 (0.9)	2229	1	50 (1.0)	4903	.72

VBDDL NCSU referral population includes all dogs' samples submitted to the VBDDL for CVBD testing from patients of the NCSU Veterinary Hospital between January 1, 2008, and December 31, 2015. The VBDDL southeast population includes all dogs' samples submitted to the VBDDL for CVBD testing from veterinary clinics located in the southeast United States (North Carolina, South Carolina, Virginia, or Tennessee) between January 1, 2008, and December 31, 2015. * indicates statistical significance, with *P* < .05.

Abbreviations: BAPGM, Bartonella Alpha Proteobacteria Growth Medium; CVBD, canine vector‐borne disease; HW, heartworm; IFA, indirect immunofluorescent assay; NCSU, North Carolina State University; PCR, polymerase chain reaction; VBDDL, Vector Borne Disease Diagnostic Laboratory.

**Table 3:** CVBD IFA and PCR results for proteinuric dogs with negative SNAP 4DX tests

CVBD pathogen	Number of positive dogs	
*Rickettsia* total	26	
IFA only	23	Includes 2 coinfection with *Mycoplasma hemocanis* (PCR)
PCR + IFA	3	Includes 1 coinfection with *Mycoplasma hematoparvum* (PCR)
*Ehrlichia* total	2	
IFA only	2	
PCR only	0	
*Mycoplasma* spp. (PCR)	5	
*M hemocanis* PCR	4	2 co‐exposures with *Rickettsia* spp. (IFA)
*M hematoparvum* PCR	1	Coinfection with *R rickettsii* (PCR) and *Rickettsia*‐positive IFA
*Babesia* total	4	
IFA only	1	
PCR only	1	*Babesia coco*
IFA + PCR	2	2 *Babesia gibsoni*
*Bartonella* total	5	
BAPGM only	2	2 *Bartonella henselae*
IFA only	2	
BAPGM + IFA	1	*B henselae* on BAPGM; 1:64 positive *B henselae* and *Bartonella vinsonii* spp. *berkhoffii* IFA
*Anaplasma* (PCR)	0	
Negative on all IFA and PCR	133	
Total positive dogs	39	

Abbreviations: CVBD, canine vector‐borne disease; IFA, indirect immunofluorescent assay; PCR, polymerase chain reaction.

There was no difference in CVBD exposure between male and female proteinuric dogs (39.4% versus 29.5%, respectively, *P* = .13). Intact dogs of either sex were more likely to have CVBD exposure (57.7%) compared to neutered dogs (31.1%; odds ratio [OR] 3.17; 95% confidence interval [CI], 1.23‐8.14), based on the multivariable logistic regression model (Table 4). Of the 2 female intact dogs, 1 was *B burgdorferi and Ehrlichia* SNAP positive, and the other was *B burgdorferi* and *D immitis* ELISA positive and *Babesia* IFA seroreactive. The mean age for both P‐CVBD+ and P‐CVBD− dogs was 8.1 years. The proportion of P‐CVBD+ and P‐CVBD− dogs did not differ among breeds. Neither age nor breed contributed significantly to the multivariable logistic regression model. The proportion of P‐CVBD+ and P‐CVBD− dogs was significantly different among seasons. The proportion of P‐CVBD+ dogs was higher in summer (44.8%) or winter (45.1%) compared to autumn (25.5%) or spring (20.4%). Presentation in the autumn or spring was independently associated with decreased probability of CVBD exposure compared to presentation in summer (OR, 0.382; 95% CI, 0.16‐0.94 and OR, 0.210; 95% CI, 0.08‐0.55, respectively).

**Table 4:** Results from multivariable logistic regression, showing ORs and 95% CIs for demographic and clinicopathologic variables that are associated with increased likelihood of CVBD exposure

		Coefficient	Standard error	OR	95% CI	*P*‐value
Intact	Versus altered					
	Intact	1.1524	0.48195	3.17	1.23‐8.14	.02*
Season	Versus summer					
	Autumn	−0.96021	0.45775	0.38	0.16‐0.94	.04*
	Winter	−0.05418	0.42023	0.95	0.42‐2.16	.90
	Spring	−1.55975	0.48755	0.21	0.08‐0.55	.001*
Alb		−0.48723	0.2468	0.61	0.38‐1.00	.05*
Creat		0.1672	0.08429	1.18	1.002‐1.39	.05*

Breed, platelet count, and serum cholesterol did not contribute significantly to the model and were removed for creation of the final model.

Abbreviations: Alb, serum albumin (g/dL); CI, confidence interval; Creat, serum creatinine (mg/dL); CVBD, canine vector‐borne disease; OR, odds ratio; Season, season of presentation.

Serum albumin concentration was lower in the P‐CVBD+ dogs compared to P‐CVBD− dogs (2.46 g/dL versus 2.67 g/dL; *P* = .04; OR, 0.61; 95% CI, 0.379‐0.996). The difference in median serum creatinine concentration between P‐CVBD+ (1.2 mg/dL) and P‐CVBD− (.8 mg/dL) dogs was not statistically significant based on initial hypothesis testing, but serum creatinine concentration did contribute significantly to the multivariable logistic regression model. Serum creatinine concentration was positively associated with increased probability of CVBD exposure (OR, 1.18; 95% CI, 1.002‐1.39; *P* = .05) indicating that for each 1 mg/dL increment increase in serum creatinine concentration, the odds of CVBD exposure increased by 1.18. There were no other differences between P‐CVBD+ and P‐CVBD− dogs for the remaining selected clinicopathologic variables, including HCT, platelet count, lymphocyte count, or serum globulin or cholesterol concentrations. Finally, there was no difference in UPC ratio between P‐CVBD+ and P‐CVBD− dogs, and UPC ratio did not contribute significantly to the multivariable logistic regression model. There were no significant interactions between age, breed, sex, or intact status and any of the selected clinicopathologic variables, indicating that the effect of the associated variables (creatinine, albumin) on CVBD pathogen exposure was not dependent on demographic factors.

The proportion of CVBD exposure was higher in the 209 proteinuric dogs than in either the VBDDL NCSU referral population or the VBDDL southeast population (34.4% in proteinuric dogs versus 25.9% in VBDDL NCSU referrals [*P* = .009] and 24.1% in VBDDL southeast [*P* < .001]; Table 2). The major differences between the proteinuric dogs and the VBDDL NCSU referral or VBDDL southeast populations were in *Rickettsia* spp. exposure (18.7% in proteinuric dogs versus 13.3% in both VBDDL NCSU referral and VBDDL southeast, *P* = .05 and *P* = .03, respectively) and *B burgdorferi* exposure (9.3% in proteinuric dogs versus 3.6% in VBDDL NCSU referral, *P* < .001; and 5.4% in VBDDL southeast, *P* = .03). There were no statistically significant differences between proteinuric dogs and either comparison population for exposure to *Ehrlichia* spp., *Babesia* spp., *Bartonella* spp., *Anaplasma* spp., or *D immitis*; except for *Ehrlichia* spp., all these pathogens were found uncommonly (<5% prevalence).

**Figure 3:**
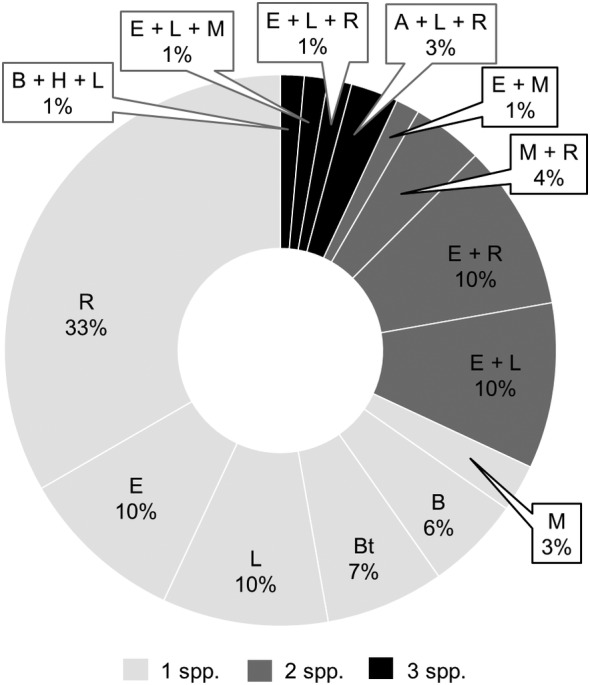
Co‐exposure between canine vector‐borne diseases in P dogs. A = *Anaplasma* spp., B = *Babesia* spp., Bt = *Bartonella* spp., E = *Ehrlichia* spp., H = heartworm disease, L = Lyme, M = hemotropic mycoplasma, R = *Rickettsia* spp. Numbers within each section show the percent of P dogs with exposure to the particular combination of species represented. Shades show the number of different species to which a dog was seroreactive/PCR positive. PCR, polymerase chain reaction

**Figure 4:**
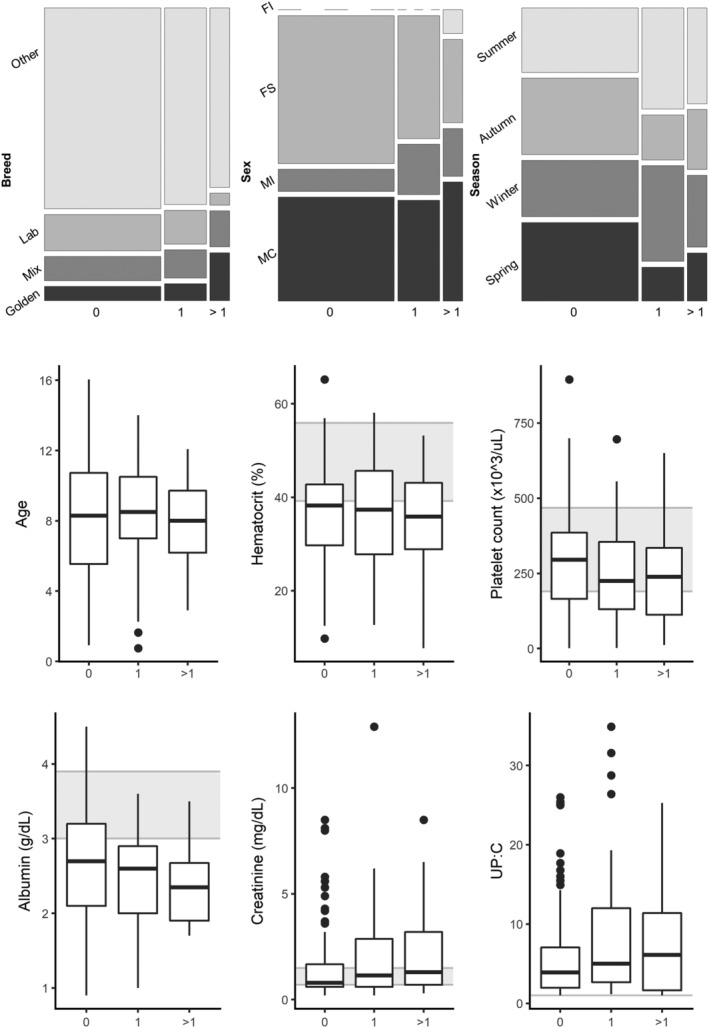
Association of demographic and selected clinicopathologic data with number of canine vector‐borne disease (CVBD) exposures/infections. The number of CVBD exposures/infections is shown on the x‐axis for all plots. For mosaic plots, the size of each box is proportional to the number of dogs in each category. For box and whisker plots, the box represents the 25th and 75th percentile, the horizontal line through the box represents the median, the whiskers represent 1.5 × IQR from the box, and the outliers are represented by dots. Gray background represents reference range for clinicopathologic data

There were 23 proteinuric dogs exposed to ≥1 CVBD pathogen (Figure 3). The most common co‐exposures were *B burgdorferi* and *Ehrlichia* spp. (9 dogs) and *Rickettsia* spp. and *Ehrlichia* spp. (8 dogs). Dogs exposed to ≥1 pathogen did not have statistically significant differences in demographic factors (age, breed, or sex) or clinicopathologic factors (albumin, creatinine, UPC ratio, HCT, or platelet count) compared to dogs with either no exposure or a single CVBD (Figure 4).

**Figure 5:**
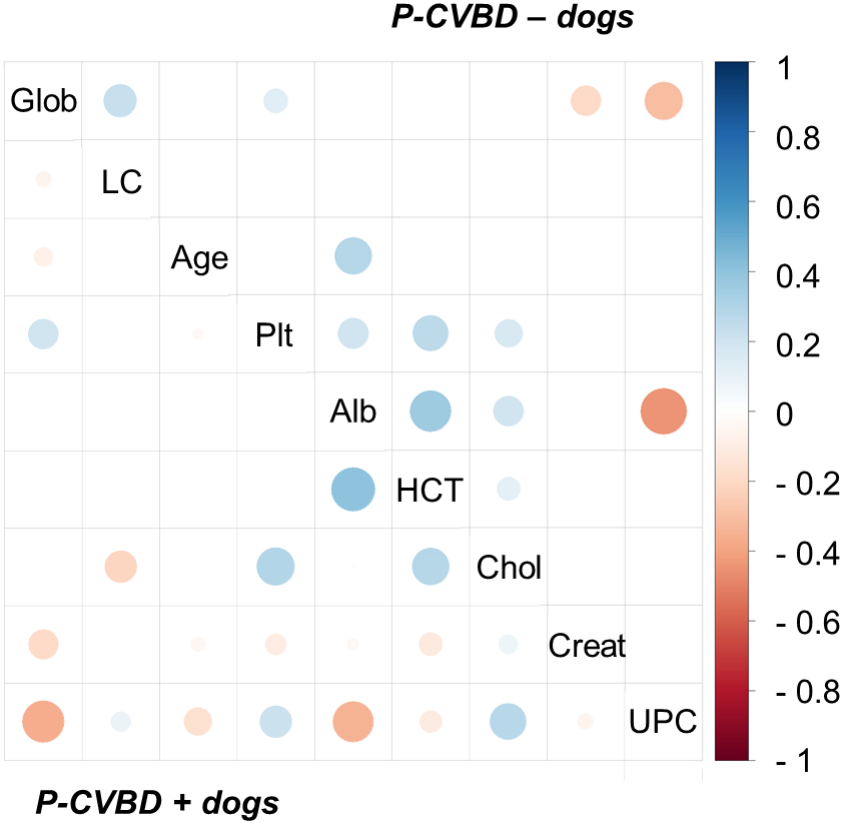
Pearson's correlations between numeric clinicopathologic variables, by group. P‐CVBD− dogs shown on the top right, and P‐CVBD+ dogs shown on the bottom left. Positive correlations are displayed in blue and negative correlations in red, with scale gradient shown on the right axis. Color intensity and the size of the circle are proportional to the correlation coefficients. Only significant correlations are shown (*P* < .05). CVBD, canine vector‐borne disease; P, proteinuria

Significant correlations were found between multiple numerical clinicopathologic variables; these correlations were similar between P‐CVBD+ and P‐CVBD− dogs (Figure 5). The strongest positive correlation was between HCT and albumin (*ρ* = 0.4 for P‐CVBD+ and *ρ* = .35 for P‐CVBD− dogs); the strongest negative correlations were between UPC ratio and albumin (*ρ* = −0.34 for P‐CVBD+ and *ρ* = −0.44 for P‐CVBD− dogs) and between UPC ratio and globulin (*ρ* = 0.36 for P‐CVBD+ and *ρ* = 0.3 for P‐CVBD− dogs). Serum albumin and globulin concentrations were not significantly correlated in P‐CVBD+ or P‐CVBD− dogs.

**Figure 6:**
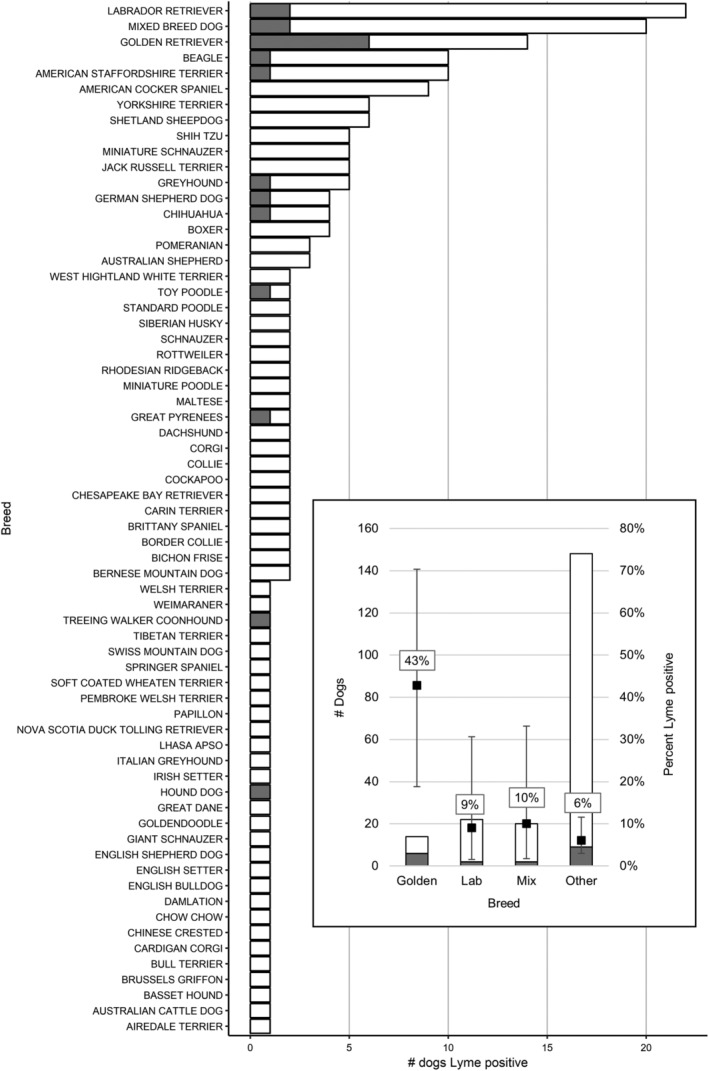
*Borrelia burgdorferi* exposure in P dogs by breed. Gray = positive Lyme SNAP test, white = negative Lyme SNAP test. Golden Retrievers are significantly overrepresented. Inset: Lyme test results summarized. The number of P dogs (left axis) and percent of dogs (right axis) seroreactive against *B burgdorferi*, by breed. Error bars represent 95% confidence intervals for percent estimates

When considering the subset of 204 proteinuric dogs tested using the SNAP 4Dx test, no significant breed differences were found between *B burgdorferi‐*exposed dogs and dogs with no evidence of exposure to *B burgdorferi*. Golden Retrievers were significantly more likely to be *B burgdorferi* seropositive, with 6 of 14 (42.9%) Golden Retrievers SNAP positive, compared to lower proportions of mixed breeds (2/20, 10%), Labrador Retrievers (2/22, 9.1%), or all other breeds combined (9/148, 6.1%; *P* = .0001; Figure 6). Based on multivariable logistic regression using demographic factors (age, breed, and sex), as well as season of presentation, only the Golden Retriever breed remained independently associated with increased likelihood of a *B burgdorferi*‐positive SNAP result (OR, 11.6; 95% CI, 2.77‐48.56; *P* < .001). Of 7 P‐CVBD+ Golden Retrievers, 6 had exposure to *B burgdorferi*; of these 7 dogs, 4 had exposure to both *B burgdorferi* and another CVBD (including *Rickettsia* spp., *Anaplasma* spp., *Ehrlichia* spp., and *Babesia* spp.). Interestingly, 12 of 19 *B*. *burgdorferi* ELISA‐positive dogs also were exposed to other CVBD pathogens. By ELISA, exposure to *B burgdorferi* was significantly associated with *Ehrlichia* spp. exposure (OR, 7.58; 95% CI, 2.15‐25.58; *P* = .0007) but not with *Anaplasma* spp. exposure, based on chi‐squared testing. Only 1 dog was ELISA‐positive for *B*. *burgdorferi* and *Anaplasma* spp.

## 4 DISCUSSION

Results obtained through our retrospective study support the possibility that exposure to ≥1 CVBD pathogens may be associated with proteinuria in dogs. Based on serology and PCR results, 34.4% (72/209) of proteinuric dogs included in our study were seroreactive to or PCR positive for at least 1 CVBD pathogen. The CVBD exposure was higher in intact dogs and lower in dogs tested in autumn or spring, and CVBD exposure was higher in dogs with lower serum albumin and higher serum creatinine concentrations. Although there was no control group of non‐proteinuric dogs with which to compare our cases, CVBD exposure (specifically exposure to SFG *Rickettsia* spp. and *B burgdorferi*) was higher in the study sample of proteinuric dogs than in the overall population of dogs tested at the VBDDL. Importantly, CVBD prevalence may have been underestimated, because not all proteinuric dogs were tested for all currently recognized CVBD pathogens by serology, PCR, or both (ie, clinicians selected tests to be performed by the laboratory). Also, the sensitivities of all CVBD testing modalities are <100%, potentially contributing to false‐negative test results. Therefore, some dogs in the P‐CVBD− group may have been misclassified as not exposed to CVBD pathogens.

*Rickettsia* and *Ehrlichia* spp. exposure were the most frequently documented CVBD exposures in proteinuric dogs, found in 18.7% and 11.7% of proteinuric dogs, respectively. *Rickettsia rickettsii*, the only currently documented pathogenic SFG rickettsial species to infect dogs in North America, causes an acute self‐limiting, doxycycline responsive, or at times fatal febrile illness in dogs.^36^ Because chronic infection with *R rickettsii* is not known to occur, the acute illness caused by this pathogen is unlikely to contribute to the development of chronic proteinuria or IMGN. Because the *Rickettsia* spp. IFA cross‐reacts with several SFG‐rickettsial organisms that are considered nonpathogenic or minimally pathogenic, it is possible that ≥1 of these *Rickettsia* spp. may in fact contribute to proteinuria. Alternatively, persistent occult infection with *R rickettsii* (or another organism that serologically cross‐reacts with *R rickettsii* antigens), which has not been documented in dogs in North America, could contribute to chronic antigen stimulation and proteinuria.^37^

In contrast to *R rickettsii*, ehrlichiosis *(E canis*, *E chaffeensis*, and *E ewingii)* results in long‐lasting intravascular infections in dogs, which could contribute to the development of proteinuria. Indeed, acute glomerulonephritis has been documented within 30 days after experimental infection with *E canis*, and nearly 75% of dogs with naturally acquired *E ewingii* in a recent retrospective study had proteinuria on urinalysis.^38^, ^39^ Future studies should further investigate the role of individual *Ehrlichia* and SFG *Rickettsia* spp. as potential causes of proteinuria in dogs.

Despite the substantial lack of *Bartonella* spp. IFA and PCR diagnostic sensitivities, 5 proteinuric dogs were exposed to a *Bartonella* spp.^40^ A potential role for *Bartonella* spp. in the pathogenesis of proteinuria has not been investigated in dogs or humans, but glomerulonephritis associated with *Bartonella* spp. endocarditis has been documented in human case reports of humans.^41^, ^42^

Among the 72 proteinuric dogs with CVBD exposure, 31.9%, had been co‐exposed to ≥2 CVBD pathogens. Some arthropods are competent vectors for transmission for >1 CVBD pathogen, and it is not unusual for dogs to be exposed to several different arthropod species simultaneously or sequentially.^16^ When compared to infection with individual pathogens, coinfections with multiple vector‐borne pathogens can contribute to complex disease expression resulting in diagnostic, therapeutic, and medical management challenges.^27^ The extent to which coinfections might contribute to proteinuria has not been systematically studied, but the high prevalence of co‐exposures and the trend toward more severe disease manifestations in the co‐exposed dogs in our study justify further study. It is possible that CVBD co‐exposures contribute to the development of proteinuria, potentially by immune complex deposition leading to glomerulonephritis or activation of inflammation leading to amyloidosis.

Collectively, large breed (Labrador Retrievers, Golden Retrievers) and mixed breed dogs comprised 28% of our proteinuria study population and 42.9% of Golden Retrievers tested were *B burgdorferi* seroreactive. Until recently, North Carolina and South Carolina have been considered nonendemic states for *B burgdorferi* transmission to dogs or humans, with Virginia being an endemic transition state for transmission of Lyme disease in humans and dogs.^43^ Based on Companion Animal Parasite Council data and publications from our laboratory, North Carolina is now an endemic state for *B burgdorferi* transmission.^26^, ^44^ In the current study, proteinuric Golden Retrievers were more likely to be *B burgdorferi* seroreactive, compared to all other breeds, and coinfection was documented in 4 of 14 Golden Retrievers. This association may support previous studies that have documented an increased risk for Golden Retrievers developing Lyme nephropathy, presumably because of a breed‐specific genetic predisposition.^45^

Because most CVBD pathogens have evolved to cause persistent intravascular or intradermal (*B burgdorferi* and perhaps *R felis*) infections in dogs, establishing causation for specific clinicopathologic abnormalities remains challenging. Proteinuric dogs with CVBD exposure had lower serum albumin concentrations and higher serum creatinine concentrations than did unexposed dogs, possibly indicating more severe disease. Although response to treatment was not evaluated in our study, antibiotic treatment in the face of seroreactivity, positive PCR results, or both should be viewed as an opportunity for treatment and intervention in a chronic disease process that may have an otherwise potentially poor prognosis. Indeed, although the International Renal Interest Society Glomerular Disease Study Group has cautioned against acceptance of a cause‐and‐effect relationship between glomerular protein loss and concurrent positive CVBD serological or PCR results, the consensus statement advises clinicians to assume (until proven otherwise) that there is a role for ≥1 CVBD pathogens as a cause of glomerular injury. Therefore, according to these consensus recommendations, directed treatment with antimicrobials should be initiated as early in the disease course as possible for dogs with identified exposure to an infectious agent and associated clinical signs.^10^

In recent years, access to rapid, in‐hospital screening tests has allowed veterinarians to assess antibodies to selected CVBD pathogens. In our study, 172 of 204 dogs were SNAP 4DX‐negative, of which 39 were determined to have CVBD exposure based on IFA or PCR testing. If only the rapid assay ELISA had been used to screen the dogs in our study, only 32 dogs would have been identified as CVBD‐positive (rather than 71), missing 55% of truly CVBD‐exposed dogs. These findings support the recommendation for more comprehensive serological and PCR testing rather than relying solely on the SNAP 4DX ELISA to facilitate an accurate microbiological diagnosis of CVBD pathogen exposure in proteinuric dogs.^32^ Previous research has shown that concurrent use of PCR and serological assays in parallel is likely to increase the detection or exposure of CVBD pathogens.^46^ Retesting, particularly once immunosuppressive treatment has been instituted, also can increase diagnostic PCR documentation of CVBD infection.^47^ Failure to diagnose a CVBD pathogen in a dog with proteinuria could result in a diagnosis of idiopathic IMGN, immunosuppressive drug treatment, and potentially an incomplete treatment response with an adverse clinical outcome.

Our study was subject to the limitations inherent in any retrospective analysis of patient medical records and results. There was no standardization of data collection or recording at the time of patient evaluations. Also, there was variability in diagnostic testing among individual dogs based on test request selection by the attending clinician. Variability in requested CVBD tests may be associated with potential clinician biases in test selection, as well as laboratory changes in test offerings over the study time frame and diagnostic improvements in assay sensitivity and specificity. For example, many seroreactive dogs did not have PCR testing requested. Therefore, the associations between CVBD and demographic and clinicopathologic variables found here reflect only associations with exposure, not necessarily active infection, with these pathogens. Because of regional variations among tick species and CVBD pathogens, the results of our study should not be extrapolated to all regions of the United States or elsewhere.

We attempted to exclude cases of renal tubular proteinuria (ie, dogs lacking serum albumin concentrations or having normal serum albumin concentration with a UPC ratio <1). This decision was based on general acceptance that UPC ratio >0.5 indicates renal proteinuria when urine lacks evidence of inflammation or macroscopic hematuria,^1^, ^2^, ^3^ and UPC ratio ≥2.0 is considered a magnitude of proteinuria sufficiently high to support pathologic proteinuria.^3^ Furthermore, all patients were evaluated by a minimum of 2 faculty clinicians and 1 resident at the time of initial diagnostic evaluation. Each case then was reviewed further by a senior internal medicine resident for inclusion in the study. Despite these attempts to include only patients with glomerular proteinuria, it is possible that we captured a subset of dogs with UPC ratio between 0.5 and 2.0 in which proteinuria was not caused by glomerular injury but was by another cause of renal proteinuria such as tubulointerstitial disease. Ultimately, without matching renal biopsy samples for each patient included, the location of renal injury leading to proteinuria cannot be definitively identified.^39^

In addition, IFA and PCR diagnostic testing for CVBD both have limitations. Both antibodies and infection can persist for variable intervals depending upon the pathogen and variations in host immune response. In addition, serology does not confirm active or persistent infection.^10^ Conversely, molecular techniques such as PCR detect active infection, but low levels of intravascular infection are common among CVBD pathogens. Therefore, PCR testing at a single time point may produce false‐negative PCR results for a dog infected with ≥1 CVBD pathogens.^10^ Because treatment history in these proteinuric dogs was unknown, there is likely also a subset of proteinuric dogs that received antibiotics before CBVD testing, potentially decreasing PCR sensitivity.

Overall, CBVD exposure, particularly exposure to *Rickettsia* and *Ehrlichia* species and *B burgdorferi*, was frequent among proteinuric dogs in our study. Establishing a direct cause‐and‐effect relationship between positive CVBD test results and proteinuria will require both controlled, prospective epidemiologic studies as well as experimental studies.^8^ With recent standardizations in renal histopathology, interpretation of renal biopsy results obtained from proteinuric dogs may allow for more precise interpretation of CVBD test results.^48^ Collectively, information derived from our study should improve the current understanding of CVBD exposure in proteinuric dogs and facilitate the design of future prospective studies targeting individual CVBD pathogens or CVBD coinfections in the development or progression of proteinuria.

## CONFLICT OF INTEREST DECLARATION

Shelly L. Vaden serves as Associate Editor for the Journal of Veterinary Internal Medicine. She was not involved in review of this manuscript.

## OFF‐LABEL ANTIMICROBIAL DECLARATION

Authors declare no off‐label use of antimicrobials.

## INSTITUTIONAL ANIMAL CARE AND USE COMMITTEE (IACUC) OR OTHER APPROVAL DECLARATION

Authors declare no IACUC or other approval was needed.

## HUMAN ETHICS APPROVAL DECLARATION

Authors declare human ethics approval was not needed for this study.
